# A novel mutation of *PAX6* in Chinese patients with new clinical features of Peters’ anomaly

**Published:** 2010-04-15

**Authors:** Xiuhua Jia, Xiangming Guo, Xiaoyun Jia, Xueshan Xiao, Shiqiang Li, Qingjiong Zhang

**Affiliations:** State Key Laboratory of Ophthalmology, Zhongshan Ophthalmic Center, Sun Yat-Sen University, Guangzhou, People’s Republic of China

## Abstract

**Purpose:**

To identify novel mutation in the *PAX6* (paired box gene 6) gene and characterize new clinical features of severe ocular malformation in a Chinese patient with Peters’ anomaly.

**Methods:**

A 10-month-old male infant, who presented with corneal opacity and nystagmus, was referred to our pediatric clinic and underwent a complete general physical and ophthalmological examination, including anterior segment and retinal evaluation with slit-lamp microscopy, an A/B ultrasonic scan, and electroretinography (ERG). Genomic DNA was prepared from venous leukocytes. The coding regions and the adjacent intronic sequence of *PAX6* were amplified by a polymerase chain reaction, and subsequently analyzed by direct sequencing. The variation detected was further evaluated in 100 controls using heteroduplex- single strand conformational polymorphism (SSCP) analysis.

**Results:**

The patient had bilateral Peters’ anomaly showing congenital nystagmus, corneal leukoma with anterior synechia, anterior polar cataract, and his pupils could not be dilated because of posterior synechia. Electroretinography (ERG) demonstrated retina hypogenesis and an A/B ultrasonic scan showed microphthalmus. A novel mutation: C.51C>A (P. N17K) was identified in *PAX6* while this mutation was absent in 100 normal controls. This mutation, which affects highly conserved amino acid, has not been previously reported.

**Conclusions:**

*PAX6* mutations cause ocular malformations that vary considerably in pattern and severity. In this study, we identified one novel mutation in *PAX6* in a patient with severe ocular clinical features of Peters’ anomaly. This finding expands the mutation spectrum in *PAX6* and enriches our knowledge of genotype-phenotype relations due to *PAX6* mutations.

## Introduction

The *PAX6* gene (OMIM 607108, paired box gene 6, a paired box transcriptional factor) is located on chromosome 11p13, consists of 14 exons, and encodes 422 amino acids [1,2] as a transcriptional regulator (expressed in the developing central nervous system and various ocular tissues), and is involved in eye morphogenesis. *PAX6* is a key regulator of eye development, and there are many well recognized ophthalmic sequelae of mutations at this locus. Human *PAX6* mutations have been associated with a variety of congenital eye malformations. Mutations in *PAX6* result mainly in aniridia [3,4]. Also, in rare cases, *PAX6* mutations cause other ocular abnormalities such as congenital cataracts [5], Peters’ anomaly [6,7], corneal dystrophy [8], autosomal dominant keratitis, foveal hypoplasia, microphthalmia, optic nerve malformations including coloboma, morning glory disc anomaly, and optic nerve hypoplasia [9,10].

Peters’ anomaly is congenital and affects the anterior segment of the eye. This disease is most often sporadic but may be recessive or occasionally dominant in inheritance. The Peters’ phenotype varies greatly. The essential feature of Peters’ anomaly is a congenital central corneal opacity. The size and density of the opacity can range from a faint stromal opacity to a dense opaque central leukoma. The phenotypes may be isolated or accompanied by other ocular malformations. Other, less frequent, ocular abnormalities occur in the microcornea, microphthalmos, cornea plana, sclerocornea, colobomata, dysgenesis of the angle and iris, ptosis, optic nerve, and foveal hypoplasia [11-13].

The mutations in *PAX6* in patients with Peters’ anomaly have been rarely reported, especially among Chinese patients. In this study, *PAX6* mutation analysis and detailed clinical evaluation were performed to identify novel mutation and characterize new clinical features of severe Peters’ anomaly ocular malformation in a Chinese patient.

## Methods

### Patients and clinical data

A 10-month-old male infant presenting with corneal opacity and nystagmus was referred to our Pediatric and Genetic Clinic in the Eye Hospital of the Zhongshan Ophthalmic Center, Guangzhou, China. Written informed consent was obtained, the study was approved by the Ethics Committee of the Zhongshan Ophthalmic Center, and was performed according to the tenets of the Declaration of Helsinki. Medical and ophthalmic histories were obtained. A complete general physical examination and a detailed ophthalmological examination, including anterior segment observation with slit-lamp microscopy and intraocular pressure (IOP) measurement, were performed by Drs. Zhang and Guo. An A/B ultrasonic scan and ERG was used to evaluate the ocular and retinal hypogenesis of dysmorphic and functional findings.

### Mutation screening

Genomic DNA was prepared from venous blood. All of the primers for *PAX6* (Table 1) were used to amplify coding exons (exon 4 to exon 14 of *PAX6*) and the adjacent intronic sequence of the two genes (NCBI human genome build 35.1, NC_000011.8 for gDNA, NM_001604.3 for cDNA, and NP_001595.2 for protein of *PAX6*). The PCR products of the exons and adjacent intronic sequences for the patient were sequenced with the ABI BigDye Terminator cycle sequencing kit v3.1 (ABI Applied Biosystems, Foster City, CA) according to the manufacturer’s recommendations, using a 3100 sequencer confirmed by ourselves. Sequencing results from patients, as well as consensus sequences of *PAX6* from the NCBI human genome database (NM_001604.3) were imported into the SeqManII program of the Lasergene package (DNAStar Inc., Madison, WI) and aligned to identify variations. Each mutation was confirmed by bidirectional sequencing. Mutation was named according to the nomenclature recommended by the Human Genomic Variation Society (HGVS).

**Table 1:** Oligonucleotides used for *PAX6* amplification.

**Exon**	**Sequences (5′-3′)**	**Annealing temperature (°C)**
4	F:TGATGCAGCTGCCCGAGGATTA	68
	R:GGGGCGAGAGGGGGTGTGAGTTA	
5	F:TTCCCCTTCCTCCTCTCCTTTCT	62.4
	R:GGGGGTCCATAATTAGCATCGTT	
6	F:GCAAGGTCAGCACAAAAATAAAT	66.5
	R:TGCCCTGGGTCTGATGGA	
7	F:AAAACGTAAGCTTGTCATTGT	58
	R:GAGAGGGTGGGAGGAGGTA	
8	F:TTCCATGCCCAAAGTGATAG	60
	R:TTCCCAGGCCAACAAAAT	
9	F:AAAATGGTGGTCAGGTAACTAAC	62.4
	R:TGAAAAGATGCCCAGAGAAATAA	
10	F:TTGGTTGGAGGTAATGGGAGTG	62.4
	R:GGAAATCAGGTGGGACAGGTTAG	
11	F:CGTGGGGAGGGCAGCAGTG	68
	R:ATGGAGCCAGATGTGAAGGAGGAA	
12	F:GAAGGGCCAAATGGAGAAGAGA	68
	R:TGCAGACACAGCCAATGAGGT	
13	F:GACTAGCTCGAGGCCCAATCTTA	64.8
	R:TTCCCTTTTCAATCCCCATCC	
14	F:TTGCTGGAAATAAAAAGTGA	58.9
	R:CGGCTCTAACAGCCATTTTT	
H-SSCP analysis of exon 5.	F:TTCTCCCTCATCTTCCTCTTCCTT	64.8
	R:GCAGTGGCCGCCCGTTGAC	

A sequence of oligonucleotides and annealing temperatures was used for exon-by-exon *PAX6* amplification. In the table, “F” indicates the forward strand and “R” indicates the reverse strand. Sequences are given in the 5′-3′ direction.

### Heteroduplex-SSCP analysis

The variation detected in the gene was further evaluated in 100 normal controls (informed consent, in accordance with the Declaration of Helsinki, was obtained from the participating individuals before the study) by using heteroduplex-SSCP analysis as previously described in the literature [14-16]. DNA fragments of 152 bp-spanning mutation sites were PCR-amplified according to Table 1. PCR product was mixed with an equal volume of gel-loading buffer (95% formamide, 20 mM EDTA, and 0.05% bromophenol blue, 0.05% xylene cyanol), and denatured at 95 °C for 5 min and immediately placed on ice for 5 min. The samples were loaded directly onto 8% polyacrylamide gels and run 8 h at room temperature at 40 w in a solution of 0.5× TBE.

## Results

**Figure 1:**
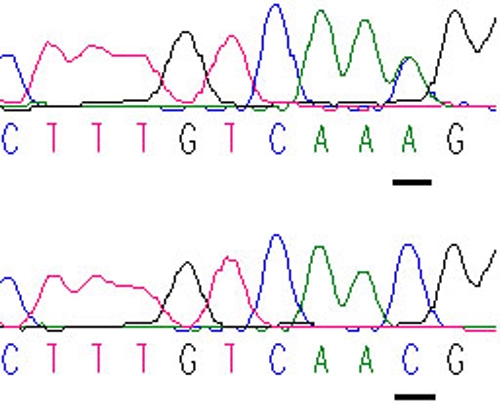
Novel *PAX6* mutation identified in exon 5 of the patient. A heterozygous mutation in *PAX6* was exclusively detected in the affected patient and the mutation was confirmed by bidirectional sequencing, which the underline indicates. The other underline shows the corresponding normal sequence from the unaffected control individual.

**Figure 2:**
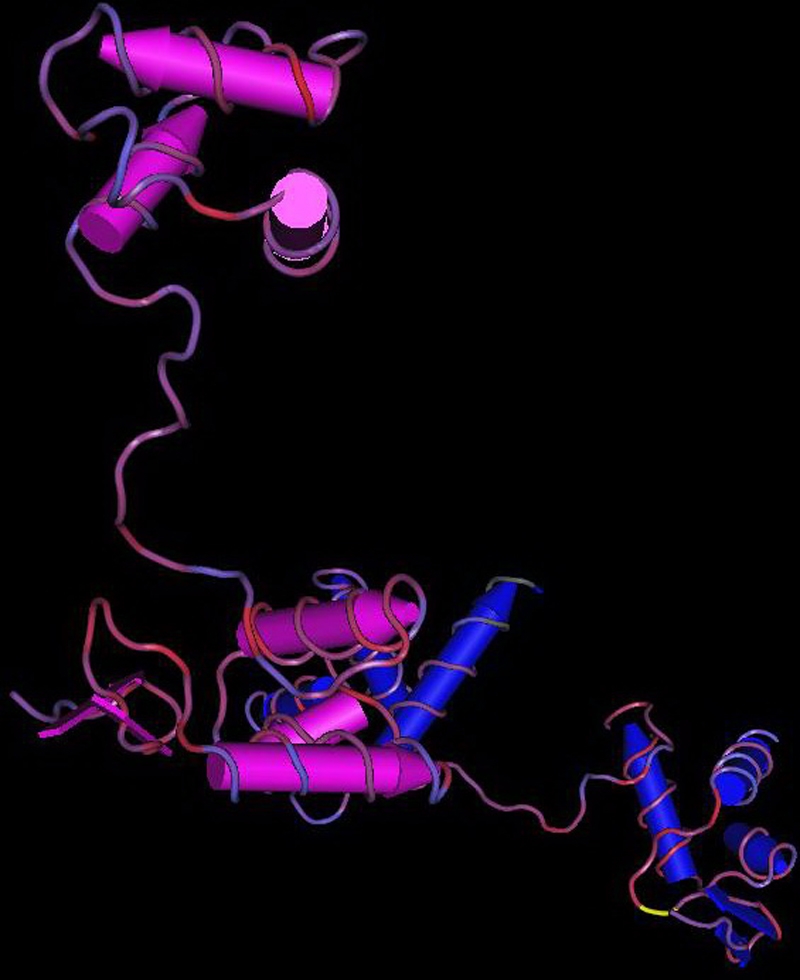
The bipartite paired DNA-binding domain of PAX6. The yellow segment represents the mutation region.

**Figure 3:**
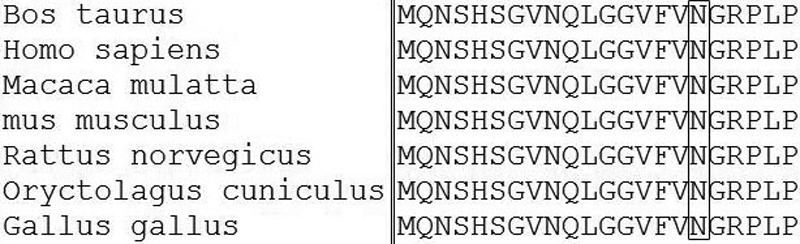
The mutation involved a highly conserved residue. As shown in the square, the threonine at position 17 is highly conserved for PAX6, which was demonstrated by analysis of 7 orthologs from different vertebrate species.

**Figure 4:**
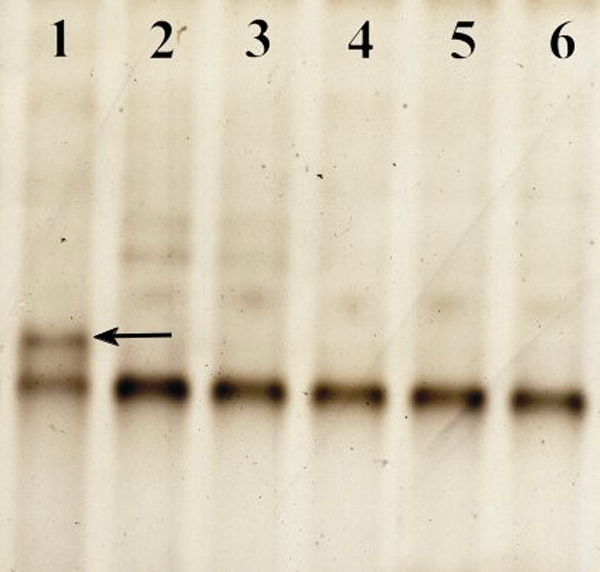
The mutation was detected by heteroduplex-SSCP analysis. Abnormal migrational SSCP band patterns in the mutation are demonstrated as compared with normal. Line 1, sample from the affected individual; lines 2 through 6, Samples from unaffected control individuals (NC). The arrow indicates the band that is different from the NC samples.

A novel heterozygous mutation, c.51C>A in *PAX6* (Figure 1), was identified. The nucleotide substitution of *PAX6* would result in replacement of Asparagine by lysine (i.e., P.Asn17Lys) at codon17. The mutation (Figure 2) is in a bipartite-paired DNA-binding domain. The p. Asn17Lys mutation identified in this study changed from an amide-type amino acid to a basic amino acid, which results in a change at the protein level with a residue weight from six to zero on Blosum 62, as well as a “probable damaging” effect by PolyPhen. Asparagine in this position was found highly conserved for PAX6 by analyzing 7 orthologs from different vertebrate species (Figure 3). This missense mutation was also analyzed in 100 unaffected control individuals (Figure 4) by heteroduplex-SSCP, but none was identified.

**Figure 5:**
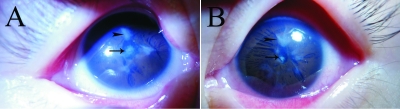
Ocular manifestations in the proband. The pictures show the aspects of the ocular anterior segment as well as of the lens of the patient. The proband had microcornea, microphthalmia, congenital corneal leukoma, iris dysplasia, and anterior polar cataracts in both eyes. In **A** (right eye) and **B** (left eye), arrowheads point to adherent corneal leucoma and arrows point to anterior polar cataracts.

**Figure 6:**
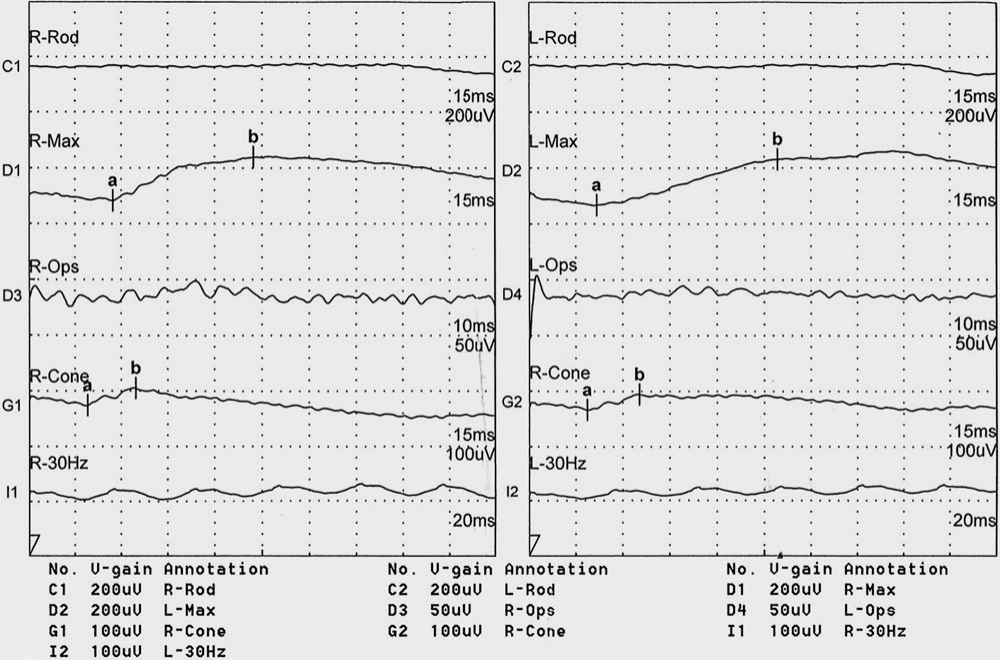
ERG examination of the patient. The results showed that the responses of the rod cells and cone cells were abnormal.

**Figure 7:**
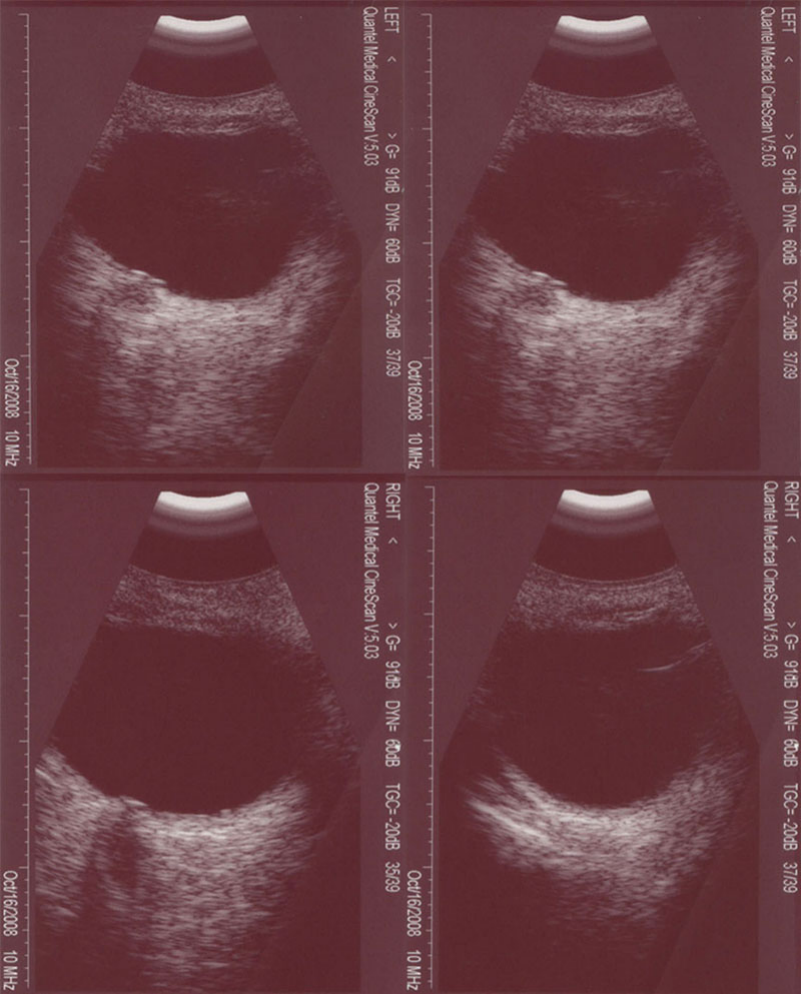
Ocular manifestations of B-scanning. B-scanning revealed the abnormal structure of the vitreous body and optic nerve.

The patient with this mutation of *PAX6* was a 10-month-old boy, born at full term after a normal delivery. His parents were not consanguineous and were healthy. Corneal opacity and nystagmus were present in both eyes of the patient at birth. Examination under anesthesia showed that his corneal diameter was small–horizontal 9.5 mm and vertical 9 mm–for both eyes, and A-scanning demonstrated microphthalmia with an axial length of 18.06 mm in the right eye and 18.07 mm in the left eye. Schiotz Tonometry showed that IOP was normal. The corneal opacity in the deep corneal stroma was irregular and fibrous adhesions between the corneal endothelial surface and the iris existed in both eyes. The anterior chamber appeared extremely shallow under microscopic examination. His iris texture was not clear, presenting in screen stencil, and the pupils could not be dilated using 0.5% tropicamide. The patient also had an anterior polar cataract with the central lens uplifted slightly. The former pole was turbid (Figure 5). The fundus could not be seen clearly by RetCom II because of the corneal opacity and the cataract. ERG examination demonstrated retinal dysphasia. A dramatic reduction of cone-specific ERG amplitude, with a more striking reduction in rod b-wave amplitude, was observed. We found no recordable rod-specific response to the ERG stimuli, while lower amplitude but normal latency of cone cells, delayed latency, and reduced amplitude of mixed response were observed (Figure 6). B-scanning showed that vitreous was opaque and an acoustic image of the binocular optic nerve showed that it was not smooth (Figure 7). A systemic examination showed that the respiratory, cardiovascular, and central nervous systems were normal in this patient.

## Discussion

Genetic analysis has detected numerous mutations in *PAX6* in sporadic cases or in families. Most previous mutations of *PAX6* have been associated with aniridia. In this study, a novel mutation (c.51C>A) in *PAX6* was identified in a Chinese patient. The signs of the patient suggest a group of developmental abnormalities in his eyes from the anterior segment to the posterior segment, which has been rarely reported. All of the clinical signs and examinations show that the boy has serious ocular eccyliosis of Peters’ anomaly [11-13].

The ERG measurements, which presented acute rod damage and attenuating function of surviving cones, reveal abnormalities in the visual cycle of the rods and cones, which may suffer comparable photoreceptor dystrophies. We focused on the development of photoreceptors to gain a better understanding of the molecular and cellular mechanisms that cause abnormal ERG. *PAX6* and *CRX* (cone-rod homeobox containing gene) are essential during early retinal development and for photoreceptor differentiation. *PAX6* expresses during the early steps of photoreceptor development, and acts as an activator of proneural genes in terms of the promotion of progenitor proliferation, maintenance of progenitor potential, and cell fate specification. To our knowledge, PAX6 protein can bind to the *CRX* promoter region in a selective manner [17], which supports its role as a direct transcriptional repressor of photoreceptor fate. However, *CRX* is a photoreceptor-specific transcription factor that plays a crucial role in the differentiation of photoreceptors, and mutations in the human homolog *CRX*, causes cone–rod dystrophy [18]. This may be a mechanism by which the specific *PAX6* mutation in our study leads to abnormal ERG. This missense mutation that produced developmental disorders of the patient in our study can be explained by the PAX6 protein structure. Many studies have determined the crystal structure of the human PAX6 paired domain with its optimal DNA-binding site [1,2,4].

The N subdomain of PAX6 uses a helix–turn–helix (HTH) unit to dock against the major groove at one end of the binding site [19]. The crystal structure reveals specific DNA contacts made by the N subdomain, which plays a dominant role in DNA binding of the intact paired domain, and provides a general model for understanding *PAX6* mutations and the protein–protein and protein–DNA interactions that are relevant for the biologic function of the paired domains. The mutation may affect the HTH structure and result in changes in structure and function. To our knowledge, the mutation in the study alters the nucleotide sequence within the N subdomain just at its optimal DNA-binding site. In PAX6, Asn-17 can directly contact DNA. The side chain of Asn-17 that lies at the DNA–protein interface in the β turn region (residues 16–19) makes a hydrogen bond with the N of guanine 9 and makes a water-mediated hydrogen bond with the same guanine [19]. In theory, the p.Asn17Lys mutation would be expected to influence the function of PAX6 protein if it is expressed. Mutation would be predicted to result in proteins with dominant negative functions or novel functions, which could have profound effects on the development of the tissues where PAX6 is normally expressed.

In terms of correlations between phenotype and mutation type, many researchers pointed out that aniridia phenotypes were caused by mutations that introduce a premature termination codon (PTC), while non-aniridia phenotypes were caused by missense mutations. They also suggested that 3′ mutations, which introduce a PTC into the *PAX6* open reading frame, do in fact yield dominant negative alleles that may cause more severe phenotypes than missense mutations [20,21]. However, in our report the missense mutation in the N subdomain can also produce serious phenotypes of Peters’ anomaly.

The potential disease-causing mutation in *PAX6* of the index cases is not very prevalent; this may be accidental due to the relatively small number of sporadic patients. It seems likely that additional genes with yet unknown functions in anterior segment development may contribute to the spectrum of Peters’ anomaly.

These results expand the mutation spectrum in *PAX6* and enrich our knowledge of genotype-phenotype relations due to the mutation. Correlating phenotype to the site of the missense mutation should be further studied. As more mutations are analyzed, it may become possible to correlate the position of a mutant, and the relative effect of the mutation on DNA structure, with the observed developmental defects.
